# A hypermorphic epithelial β-catenin mutation facilitates intestinal tumorigenesis in mice in response to compounding WNT-pathway mutations

**DOI:** 10.1242/dmm.019844

**Published:** 2015-11-01

**Authors:** Michael Buchert, Franziska Rohde, Moritz Eissmann, Niall Tebbutt, Ben Williams, Chin Wee Tan, Alexander Owen, Yumiko Hirokawa, Alexandra Gnann, Gertraud Orend, Gayle Orner, Rod H. Dashwood, Joan K. Heath, Matthias Ernst, Klaus-Peter Janssen

**Affiliations:** 1 Walter and Eliza Hall Institute, Parkville, Victoria 3052, Australia; 2 Department of Medical Biology, University of Melbourne, Parkville, Victoria 3052, Australia; 3 Olivia Newton-John Cancer Research Institute, Heidelberg, Victoria 3084, Australia; 4 School of Cancer Medicine, La Trobe University, Heidelberg, Victoria 3084, Australia; 5 Department of Surgery, Klinikum rechts der Isar, Technische Universität München, 81675 Munich, Germany; 6 Inserm U1109, MN3T team, 3 Av. Molière, Strasbourg 67200, France; 7 LabEx Medalis, Université de Strasbourg, Strasbourg 67200, France; 8 Fédération de Médecine Translationnelle de Strasbourg (FMTS), Strasbourg 67200, France; 9 University of Wisconsin, Madison, WI 53706, USA; 10 Texas A&M Health Science Center, Center for Epigenetics and Disease Prevention, Houston, TX 77030-3303, USA

**Keywords:** Colorectal cancer, Mouse models, GpA33, Inflammation

## Abstract

Activation of the Wnt/β-catenin pathway occurs in the vast majority of colorectal cancers. However, the outcome of the disease varies markedly from individual to individual, even within the same tumor stage. This heterogeneity is governed to a great extent by the genetic make-up of individual tumors and the combination of oncogenic mutations. In order to express throughout the intestinal epithelium a degradation-resistant β-catenin (*Ctnnb1*), which lacks the first 131 amino acids, we inserted an epitope-tagged ΔN(1-131)-β-catenin-encoding cDNA as a knock-in transgene into the endogenous *gpA33* gene locus in mice. The resulting *gpA33*^ΔN-Bcat^ mice showed an increase in the constitutive Wnt/β-catenin pathway activation that shifts the cell fate towards the Paneth cell lineage in pre-malignant intestinal epithelium. Furthermore, 19% of all heterozygous and 37% of all homozygous *gpA33*^ΔN-Bcat^ mice spontaneously developed aberrant crypt foci and adenomatous polyps, at frequencies and latencies akin to those observed in sporadic colon cancer in humans. Consistent with this, the Wnt target genes, *MMP7*  and Tenascin-C, which are most highly expressed in benign human adenomas and early tumor stages, were upregulated in pre-malignant tissue of *gpA33*^ΔN-Bcat^ mice, but those Wnt target genes associated with excessive proliferation (i.e. *Cdnn1*, *myc*) were not. We also detected diminished expression of membrane-associated α-catenin and increased intestinal permeability in *gpA33*^ΔN-Bcat^ mice in challenge conditions, providing a potential explanation for the observed mild chronic intestinal inflammation and increased susceptibility to azoxymethane and mutant *Apc*-dependent tumorigenesis. Collectively, our data indicate that epithelial expression of ΔN(1-131)-β-catenin in the intestine creates an inflammatory microenvironment and co-operates with other mutations in the Wnt/β-catenin pathway to facilitate and promote tumorigenesis.

## Abstract (precis)

**Summary:** The gpA33ΔN-Bcat mice provide a model that better mimics some aspects of sporadic colon cancer induction in humans in terms of tumor multiplicity and latency and site-specific (i.e. colon) tumor occurrence that coincides with upregulation of markers for human colorectal cancer progression.

## INTRODUCTION

Colorectal tumorigenesis is promoted by chronic inflammation of the intestine, and individuals suffering from Crohn's disease or ulcerative colitis have an increased risk of developing colorectal cancer (CRC). The canonical Wnt/β-catenin signaling pathway is aberrantly activated in the majority of colorectal cancers. Mutations of the *APC* (adenomatous polyposis coli) gene are the most common form of genetic alteration in CRC and represent the earliest detectable genetic change in tumorigenesis (Powell et al., 1992; Jen et al., 1994; Smith et al., 1994). Most of the tumor-suppressing functions of *APC* are attributed to its capacity for negative regulation of β-catenin, a central component of the canonical Wnt/β-catenin signaling pathway (Polakis, 1997, 2000). Accordingly, *APC* impairment mutations, epigenetic silencing (Gay et al., 2012; Dimberg et al., 2013; Qiu et al., 2014) or amino terminal mutations in *CTNNB1* that result in excessive stabilization and nuclear accumulation of β-catenin result in excessive TCF/LEF (T-cell factor/lymphoid enhancer binding factor)-dependent transcription and associated neoplastic transformation and intestinal adenoma formation. Besides its role in the canonical Wnt/β-catenin pathway, APC also regulates cell migration, adhesion, chromosome segregation, spindle assembly and apoptosis (Hanson and Miller, 2005; Dikovskaya et al., 2007). Meanwhile, a pool of β-catenin localizes at the cell membrane to maintain integrity of cell-cell adherens junctions by linking E-cadherin to α-catenin and the actin cytoskeleton (Lilien and Balsamo, 2005).

The various *APC* truncation mutations identified suggest molecular complexity of the mechanism(s) by which deregulated Wnt/β-catenin signaling drives intestinal tumor formation. For instance, *Apc*^1638N^ mice express undetectable levels of C-terminal truncated Apc protein and carry five or six tumors by 10 months of age, whereas *Apc*^Min^ mice develop more tumors with a shorter latency period, which is preceded by the obligatory loss of *Apc* heterozygosity (Fodde et al., 1994; Luongo et al., 1994). Likewise, enforced expression of an amino-terminally truncated β-catenin that lacks the 76 amino acids encoded by exon 3 leads to the formation of numerous adenomatous polyps in the small intestine and some microadenomas in the colon (Harada et al., 1999; Leedham et al., 2013). However, calbindin promoter-dependent overexpression of the more severe ΔN131β-catenin truncation mutant results in multifocal dysplastic lesions in the small intestine after 3-4 weeks, with mice succumbing prematurely to polycystic kidney disease (Romagnolo et al., 1999). A shared feature of the above models is their propensity to develop the majority of tumors in the small intestine, rather than a few early tumors in the colon, like the majority of humans of age >50 years, which bear the risk of ultimately developing into sporadic metatstatic CRC. In an effort to address these shortcomings, we developed a novel knock-in mouse model that exploits the intestine-specific *gpA33* gene locus to enforce expression of a ΔN131β-catenin-encoding transgene throughout the epithelial mucosa. Surprisingly, the corresponding *gpA33*^ΔN-Bcat^ mice show a mild decrease in epithelial barrier function associated with elevated expression of inflammatory cytokines prior to the spontaneous development of a small number of tumors, primarily colonic, in aging mice. We therefore propose that *gpA33*^ΔN-Bcat^ mice can serve to investigate the compounding pathophysiological consequences of reduced barrier function, ensuing inflammation and oncogenic driver mutations in the colon, as well as serving as a model for the development of new chemopreventive and/or therapeutic strategies (Orner et al., 2002).

**TRANSLATIONAL IMPACT**

**Clinical issue**

Colon cancer is the second leading cause of cancer mortality in many industrialized countries. Activation of the Wnt/β-catenin pathway occurs in the vast majority of colorectal cancers. Colorectal tumorigenesis is promoted by chronic inflammation of the intestine, and individuals suffering from Crohn's disease or ulcerative colitis have an increased risk of developing colorectal cancer. Genetically engineered mice are invaluable tools for deciphering the mechanisms underpinning cancer development and provide a means to test new anti-cancer drugs.

**Results**

To mimic human sporadic colon cancer in mice, a cDNA encoding truncated ΔN(1-131)β-catenin was introduced as a knock-in transgene into the intestinal gene-specific *gpA33* locus of C57Bl/6J mice and combined with various oncogenic driver mutations. The resulting *gpA33*^ΔN-Bcat^ mice show increased constitutive Wnt/β-catenin pathway activation in the intestinal epithelium and spontaneously develop aberrant crypt foci and adenomatous polyps at frequencies and latencies akin to those observed in sporadic colon cancer in humans. Consistent with this, the Wnt target genes *MMP7* and Tenascin-C, which are expressed at high levels in benign human adenomas and early colon cancer stages, were upregulated in pre-malignant tissue of *gpA33*^ΔN-Bcat^ mice. Moreover, intestinal permeability in *gpA33*^ΔN-Bcat^ mice was increased, resulting in mild chronic intestinal inflammation and increased susceptibility to azoxymethane-induced and mutant-*Apc*-dependent tumorigenesis.

**Implications and future directions**

The *gpA33*^ΔN-Bcat^ mice provide a model that better mimics some aspects of sporadic colon cancer induction in humans, including tumor multiplicity and latency and site-specific (i.e. colon) tumor occurrence; in addition, these mice show upregulation of markers of colorectal cancer progression in humans. Therefore, the *gpA33*^ΔN-Bcat^ mice are likely to be useful for functional assessment and identification of mutations that co-operate with canonical Wnt/β-catenin signaling during the initiation of adenoma formation. In addition, this approach is likely to identify components that might constitute new pharmacological targets for the treatment and prevention of sporadic colon cancer in humans.

## RESULTS

### ΔN-Bcat is expressed in intestinal epithelial cells of *gpA33*^ΔN-Bcat^ mice

The gpA33 antigen is a glycosylated transmembrane protein that is expressed specifically in the intestinal epithelium (Catimel et al., 1996; Heath et al., 1997; Johnstone et al., 2000). We exploited the endogenous *gpA33* gene locus to drive intestine-specific expression of an N-terminal deletion mutant of β-catenin (ΔN-Bcat) encoded by a bicistronic gpA33-IRES-ΔN-βcatenin RNA (Fig. S1A,E). For this, a cDNA encoding ΔN(1-131)β-catenin, in which a FLAG epitope replaced the first 131 amino terminal amino acids, was inserted in the 3′ untranslated region (UTR) of the *gpA33* antigen gene locus (Orner et al., 2002). As *gpA33* is also transcribed in embryonic stem cells, we blocked the transcription of *ΔN-Bcat* by a lox(P)-flanked neo cassette in the corresponding *gpA33*^Neo^ (henceforth referred to as A33Neo) mouse strain. Transcriptional activation of the silent *ΔN-Bcat* transgene was achieved by excising the lox(P)-flanked neo cassette in the germline to yield the *gpA33*^ΔN-Bcat^ (henceforth referred to as Bcat) strain (Fig. S1A).

To verify tissue-specific expression of *ΔN-Bcat*, we performed RT-qPCR analysis on different tissues in A33Neo and Bcat mice. We detected ΔN-βcatenin mRNA in the small and large intestines of Bcat mice but not in liver or in intestines of A33Neo mice (Fig. S1C). Transcript levels of the truncated β-catenin were higher in the large intestine as predicted from the rostrocaudal expression gradient of endogenous gpA33 (Fig. S1D). We confirmed by northern blot and anti-FLAG immunoprecipitation analysis that *ΔN-Bcat* transgene expression occurred in a gene dose-dependent manner (Fig. S1E,K) and that it did not affect expression of endogenous β-catenin in Bcat mice (Fig. S1B)*.* We also ascertained nuclear accumulation of ΔN-βcatenin by subcellular fractionation of intestinal lysates from Bcat mice (Fig. S1H). As predicted from the introduced truncation mutations, immunoprecipitation with an anti-E-cadherin antibody confirmed that ΔN-βcatenin retained the capacity to bind to E-cadherin at the membrane (Fig. S1I). The expression level of the mutant ΔN-βcatenin was approximately 50% compared with endogenous β-catenin in total lysate and further reduced to about 30% in the nuclear compartment (Fig. S1F-H).

### Increased number of Paneth cells in Bcat mice

**Fig. 1.**
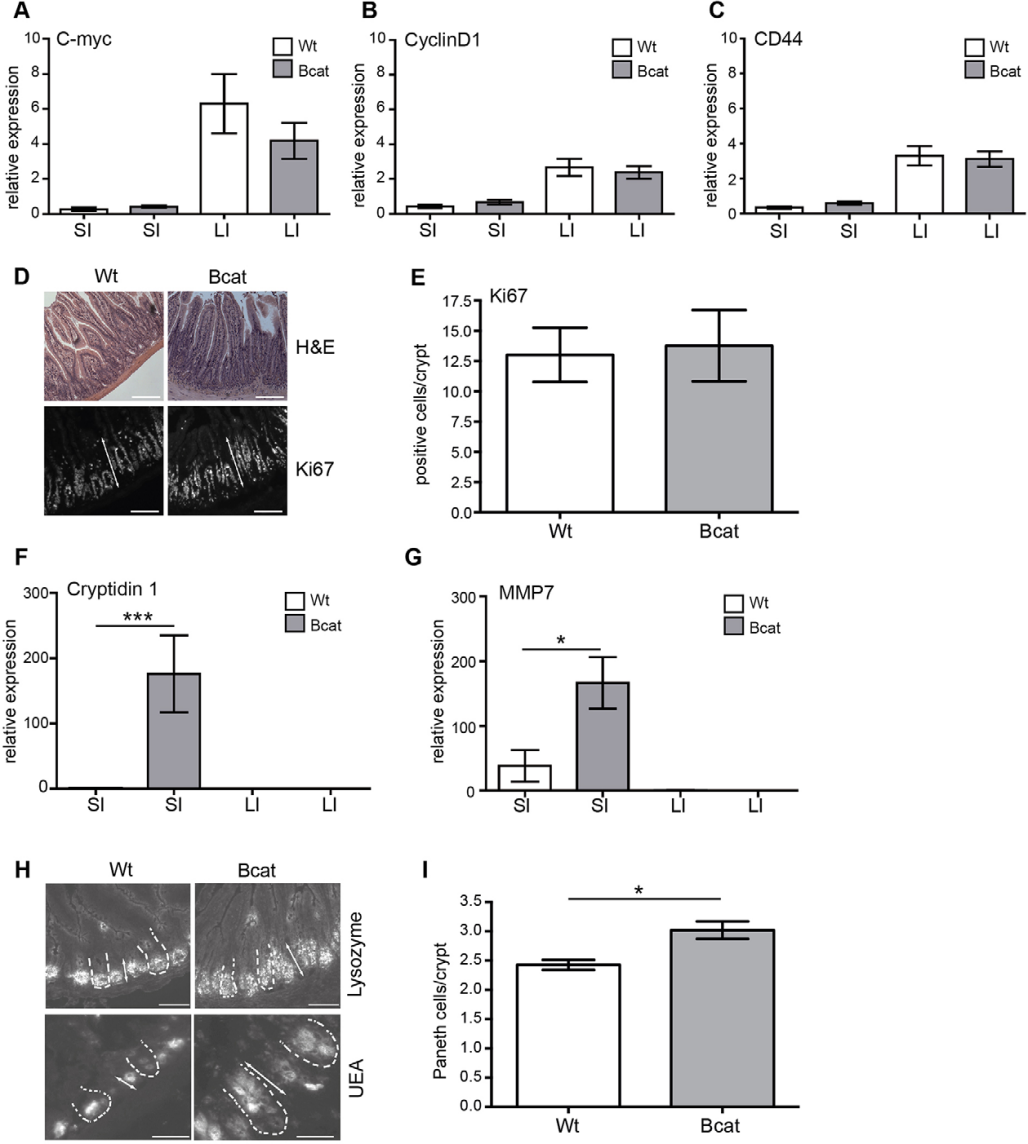
**Only some Wnt target genes are induced in pre-malignant intestinal tissue of Bcat mice.** (A-C) mRNA expression determined by quantitative real-time PCR in small (SI) and large intestine (LI) from wild-type (Wt; *n*=5) and Bcat (*n*=12) mice. Expression was normalized to the median expression of all Wt SI and LI samples, respectively. All measurements were done in duplicate. (D) Hematoxylin and eosin (H&E) and Ki67 staining of SI from Wt and Bcat mice (100× magnification, scale bar=100 µm). (E) Quantification of Ki67^+^ cells in the intestine of Wt and Bcat mice normalized per fully visible crypt. (F,G) Cryptidin 1 and *MMP7* mRNA expression in the SI and LI of Wt and Bcat mice. mRNA expression is increased in SI in Bcat mice in comparison to Wt mice but absent in the large intestine. (*P<0.05, ***P<0.001). (H) Staining for lysozyme (100× magnification; scale bar=100 µm) and Ulex Europaeus Agglutinin lectin (UEA; 200× magnification, scale bar=50 µm) in the SI of Wt and Bcat mice. The dashed lines indicate the outline of individual intestinal crypts. (I) Quantification of Paneth cells in the SI of Wt and Bcat mice (*P<0.05; *n*=3 mice per genotype; 20 fully visible crypts per mouse were counted).

To evaluate the capacity of ΔN-βcatenin to regulate Wnt target genes, we performed RT-qPCR analysis on small and large intestinal tissue of wild-type (Wt) and Bcat mice. Although expression of the constitutive active Δ-exon 3 β-catenin mutant results in elevated expression of the prototypical Wnt target genes *myc*, cyclinD1 and *CD44*, expression of these genes remained unaffected in Bcat mice (Fig. 1A-C). In agreement with this, we found similar staining patterns for the proliferation marker Ki67 in the crypts of Lieberkühn in Bcat and Wt mice (Fig. 1D,E). However, in the small intestine of Bcat mice we detected a significant increase of the Paneth-cell-specific transcripts encoding matrix metalloproteinase 7 (MMP7) and cryptidin 1, and this coincided with a significant expansion in the staining zone of Ulex Europaeus Agglutinin lectin (UEA) and lysozyme^+^ cells (Fig. 1F-I). Collectively, these observations indicate a gradient whereby limited overexpression of a stabilized ΔN-βcatenin can selectively shift the cell fate towards the Paneth cell lineage as previously described (van Es et al., 2005; Andreu et al., 2008), but without increasing the rate of mucosal renewal associated with excessive activation of the Wnt target genes *myc* and cyclinD1.

### Inflammatory cytokines in Bcat mice

**Fig. 2.**
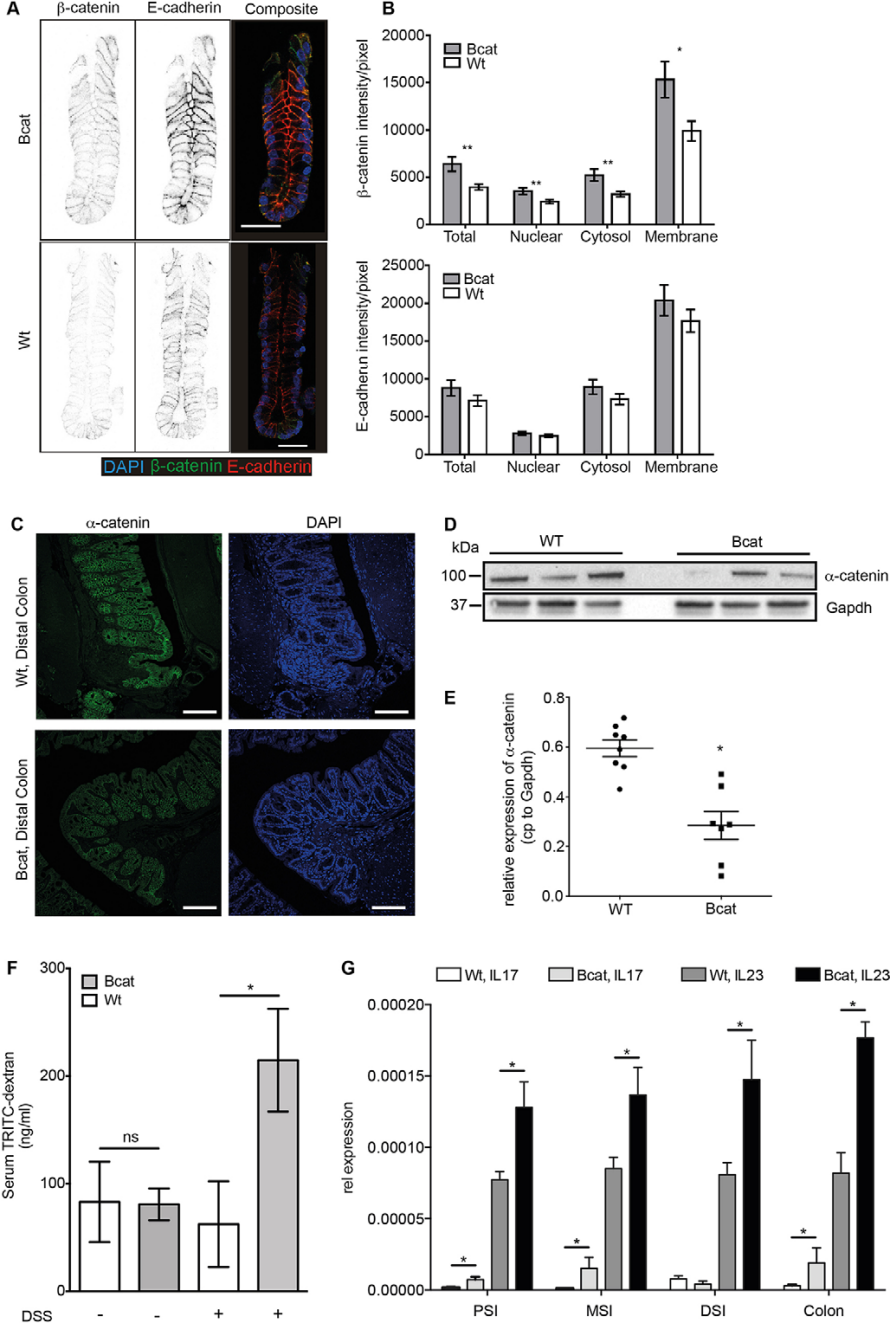
**Intestinal permeability defect in Bcat mice is associated with mislocalization of membrane-associated proteins.** (A) β-catenin and E-cadherin staining on isolated colonic crypts from Bcat and Wt mice. In the composite image, β-catenin is stained green, E-cadherin red and DAPI-labeled cell nuclei blue (scale bar=30 µm). (B) Quantification of staining intensity of β-catenin (top panel) and E-cadherin (bottom panel) in the indicated subcellular compartments (*P<0.05, **P<0.01, single-factor ANOVA, *n*=15 Bcat and *n*=24 Wt mice). (C) Confocal immunofluorescence images of α-catenin (green) in distal colon of Bcat and Wt mice. DAPI (blue) labels nuclei (scale bar=100 µm). (D) Western blot analysis of α-catenin protein expression in colonic shake preparations of Wt and Bcat mice. Shown is a representative western blot from one of three independent experiments. Each lane represents one mouse. Gapdh was used as loading control. (E) Scatter plot showing the expression levels of α-catenin protein normalized to Gapdh in colonic shake preparations of Wt and Bcat mice (*P<0.05; *n*=9 mice per genotype). (F) Intestinal permeability determined by the absorbance of TRITC-dextran in the serum 4 h after oral gavage of TRITC-dextran (4 kDa) in Wt and Bcat mice. A cohort of mice was also challenged with 2% DSS in the drinking water for 16 h prior to the TRITC-dextran gavage (*P<0.05, *n*=5-8 mice per genotype). (G) mRNA expression of IL-17 and IL-23 cytokines in proximal (PSI), middle (MSI) and distal small intestine (DSI) as well as colon of Wt and Bcat mice (*P<0.05, *n*=3 mice per genotype).

Expression of (ΔN28-134)β-catenin results in loss of intercellular adhesiveness (Oyama et al., 1994) in the human gastric signet ring cell carcinoma cell line HSC-39 through impaired interaction between mutant β-catenin, α-catenin and E-cadherin. However, we found no overt mislocalization of membrane-bound E-cadherin in cultures of intact colonic crypts isolated from Bcat mice, consistent with our biochemical analysis and the prediction that ΔN-Bcat protein retains its E-cadherin interaction domain (Fig. 2A,B; Fig. S1I). We detected increased β-catenin expression in the nucleus, cytosol and membrane fraction of isolated intestinal epithelium (Fig. 2B) that was associated with the expected reduction of α-catenin protein levels, as determined by western blotting and immunofluorescent staining  in the colon of Bcat mice (Fig. 2C-E), suggesting that ΔN-Bcat interfered with the formation of a functional α-catenin/β-catenin/E-cadherin complex at the membrane. To investigate the likely physiological consequences of aberrant formation of such membrane complexes, we measured intestinal permeability in Bcat mice using TRITC-labeled dextran (TD) gavage and observed leakage from the intestinal lumen into the circulation through quantification of serum TD 4 h later*.* Whereas the permeability to 4 kDa TD remained comparable between unchallenged Wt and Bcat mice, we noted increased leakiness of the intestinal mucosa of Bcat mice when challenged by limited exposure to the luminal irritant dextran sulfate sodium (DSS) for 16 h (Fig. 2F). Following administration of 2% DSS in drinking water, Bcat mice demonstrated a significant increase in serum TD, suggesting an underlying sensitivity of their intestinal mucosa to reduced barrier function. However, the increase in the inflammatory cytokines interleukin-17 (IL-17) and interleukin-23 (IL-23; Fig. 2G) alongside the reduced expression of α-catenin (Fig. 2C-E), present already in the unchallenged colons of Bcat mice, implies influx of innate immune cells (Grivennikov et al., 2012), possibly as a result of increased permeability to molecules smaller than TD.

### Spontaneous, sporadic colonic tumor formation in Bcat mice

**Fig. 3.**
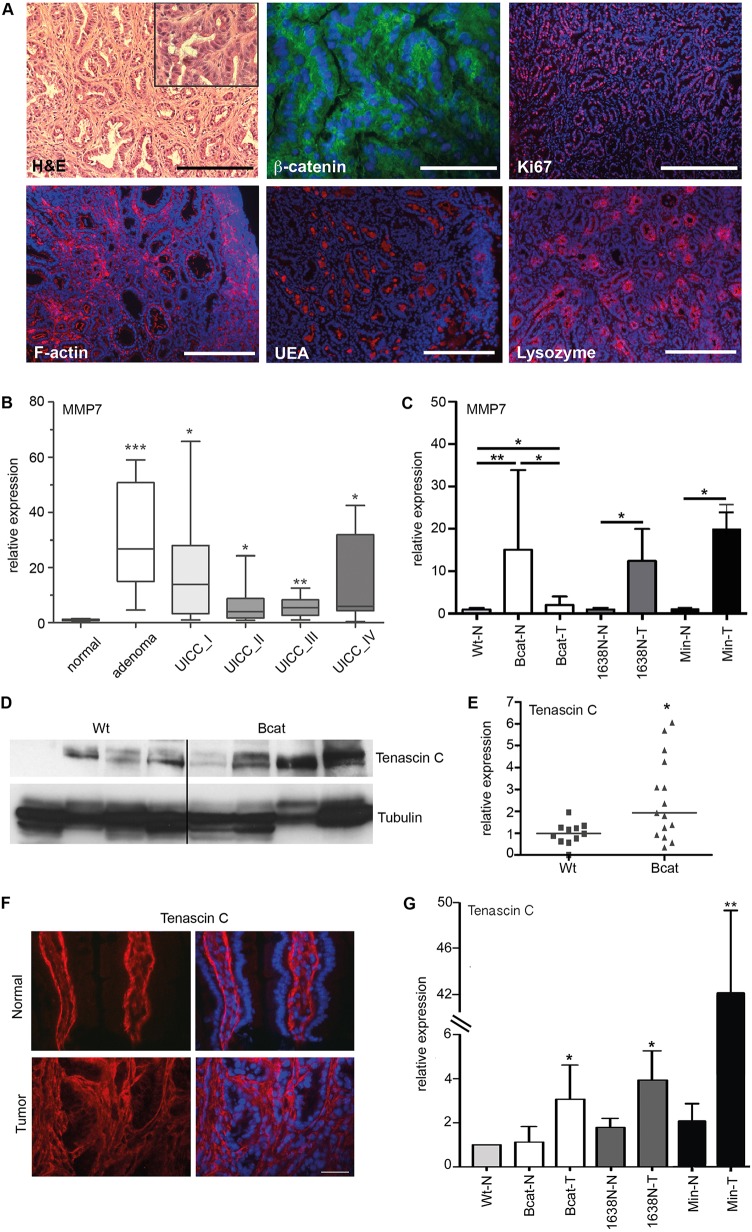
**MMP7 is upregulated in normal epithelium of Bcat mice and early stage human colon cancer.** (A) Spontaneously arising tumors in the small intestine of Bcat mice show well-differentiated to high-grade adenoma (50× magnification, scale bar=200 µm; inset: 200× magnification). Immunofluorescence staining for β-catenin, the proliferation marker Ki67, F-actin (TRITC-phalloidin), and the Paneth cell markers UEA and lysozyme. Cell nuclei are stained blue with DAPI (β-catenin: 400× magnification, scale bar=25 µm; all others: 100× magnification, scale bar=100 µm). (B) *MMP7* RNA expression in human colon cancer specimen in normal mucosa (*n*=8), benign adenomas (*n*=9), locally restricted early stage carcinoma (UICC I; *n*=11), carcinoma UICC stage II (*n*=10), carcinoma UICC stage III (*n*=8) and carcinoma UICC stage IV (*n*=13). The RT-qPCR data were normalized to expression of hypoxanthine phosphoribosyltransferase (HPRT) (*P<0.05, **P<0.01, ***P<0.001, Student's unpaired *t*-test). (C) RT-qPCR analysis of *MMP7* gene expression in normal (N) and tumor (T) tissue from mice of the indicated genotypes (*P<0.05, **P<0.01, *n*≥3, Student's *t*-test). (D,E) Western blot analysis and associated quantification of Tenascin-C protein expression in non-neoplastic small intestine of Wt and Bcat mice. Each lane represents one mouse (*P<0.05; *n*=4 mice per genotype). (F) Immunofluorescent staining with a specific anti-Tenascin-C antibody in normal and tumoral intestine of Bcat mice. Cell nuclei are stained blue with DAPI (100× magnification, scale bar=100 µm). (G) RT-qPCR analysis of Tenascin-C expression in normal (N) and tumor (T) tissue from mice of the indicated genotypes (*P<0.05, **P<0.01, *n*≥3, Student's *t*-test).

Although stronger alleles of mutant β-catenin rapidly induce extensive epithelial hyperproliferation and formation of up to several hundreds of adenomas primarily in the small intestine of mice (Harada et al., 1999; Leedham et al., 2013), tumor formation in these mice does not reflect tumor latency, multiplicity or the site where the majority of adenomas arise in humans, from which CRC ultimately develops. We therefore determined spontaneous tumor occurrence in old (8-24 month) Bcat mice and found that 22 of 54 (37%) homozygous mice carried macroscopically visible colonic tumors at an average rate of 1.7 tumors per mouse. Predictably, spontaneous tumor formation was less in heterozygous mice, in which only 6 of 31 (19%) mice showed macroscopically visible adenomas (1.5 tumors per mouse; Table S1). Meanwhile, none of the age-matched Wt littermates (*n*=18) showed any sign of intestinal lesions. Most of the detected lesions in homozygous Bcat mice were relative small colonic adenomas (<1.0 mm); however, in old mice (>22 months) we also occasionally detected larger adenomas (1.9-2.7 mm diameter). Histopathological analysis revealed that all tumors in Bcat mice corresponded to hyperproliferative tubular adenomas with equally sized cell nuclei and a prominent desmoplastic reaction (Fig. 3A). Furthermore, β-catenin was diffusely localized throughout the cytosol and nuclei of tumor cells, but clearly remained associated with the plasma membrane in adjacent normal epithelium. Meanwhile, non-uniform organization of F-actin was observed throughout the tumors, and these lesions also stained strongly for UEA and lysozyme, and prominently for Ki67 (Fig. 3A). Consistent with this, the Paneth cell marker *MMP7*, which was among the few Wnt target genes deregulated in the normal epithelium of Bcat mice (Fig. 1G), was already significantly upregulated in benign human colonic adenoma (Fig. 3B). Like MMP7, Tenascin-C, another Wnt target gene, is also upregulated in the normal mucosa of Bcat mice (Fig. 3C-E), and this persists in the tumors of these mice and in human colorectal cancer samples (*n*=31; Fig. S2A,B). Consistent with the expression of MMP7 and Tenascin-C being highly sensitive to Wnt-pathway activation, we also detected upregulation of these genes within the tumors of *Apc* mutant mice (Fig. 3C,G).

### Bcat mice are sensitized to azoxymethane-induced tumorigenesis

**Fig. 4.**
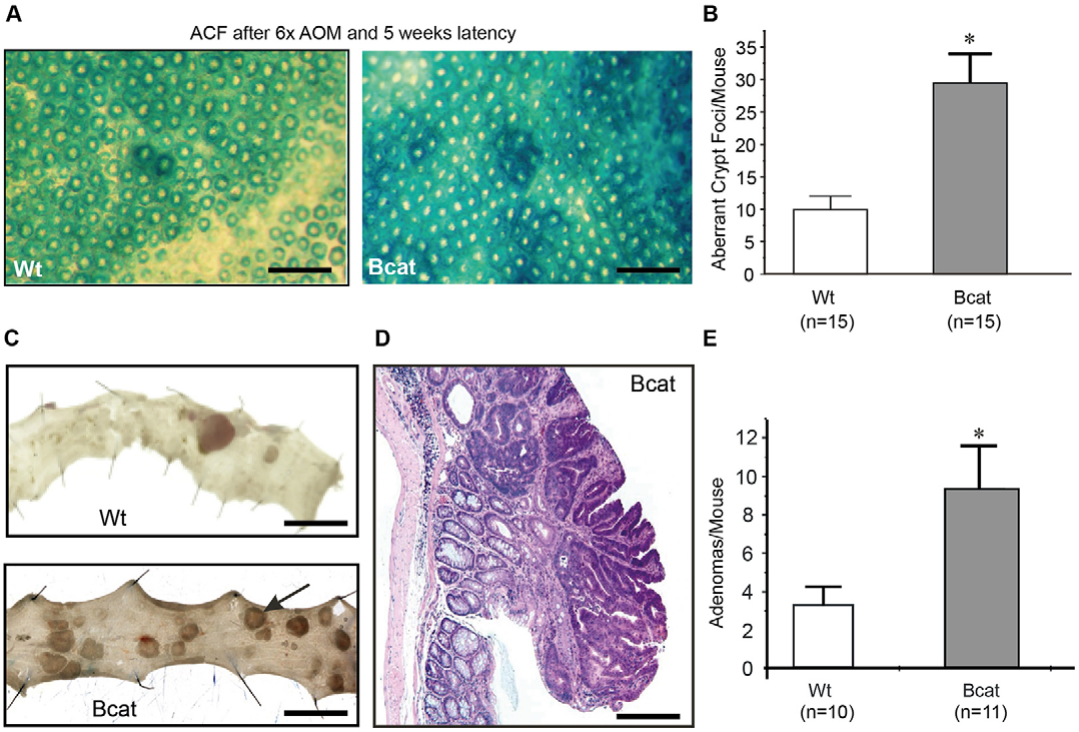
**The intestinal epithelium of Bcat mice has increased susceptibility to mutagen-induced carcinogenesis.** (A) Representative methylene blue-stained large intestine of Wt and Bcat mice 5 weeks after the last of six consecutive azoxymethane (AOM) injections (scale bar=400 µm). (B) Enumeration of aberrant crypt foci in the colonic epithelium of Wt and Bcat mice (mean±s.d., *n*=15 mice, *P<0.05). (C) Photomicrographs of longitudinally opened and pinned out colons from Wt and Bcat mice 16 weeks after last injection of AOM (scale bar=1 cm). (D) Hematoxylin and eosin stain of a representative colonic adenoma excised from a Bcat mouse 16 weeks after the last AOM injection (scale bar=20 µm). (E) Enumeration of macroscopically visible adenomas in AOM-challenged Wt and Bcat mice (mean±s.d., *n*=10-11 mice, *P<0.05).

To establish whether expression of the *ΔN-Bcat* transgene sensitized mice to chemical tumorigenesis, we challenged Bcat mice with the organotropic carcinogen azoxymethane (AOM). Mice were then analysed 5 and 12 weeks after the last of six consecutive AOM injections, and colons were stained in methylene blue to identify aberrant crypt foci (McLellan and Bird, 1988). Compared with Wt mice, Bcat mice harbored more aberrant crypt foci and more adenomatous polyps (Fig. 4A-C,E); however, these consistently remained well differentiated (Fig. 4D).

In order to gain insights into the molecular events underpinning the co-operation between the *ΔN-βcatenin* allele and AOM, we determined the nucleotide sequences of the mutagen hotspots in the endogenous *Kras* and *ctnnb1* genes. We found similar frequencies of activating missense mutations affecting codons 12, 13 and 61 of the *Kras* gene in tumors of Wt and Bcat mice. However, only tumors of Bcat mice (4 of 24) carried missense mutations in the *ctnnb1* codons that substituted the regulatory serine residues at positions 37 and 38 (Table S2) to result in a stabilized, more active form of β-catenin*.* In order to determine whether AOM also introduced functionally equivalent nonsense mutations in the *Apc* gene that result in a truncated, less stable or non-functional Apc protein, we stained tumor sections with an antibody that specifically recognizes a C-terminal epitope of the Apc protein. We observed weaker Apc staining in a much larger proportion of adenomas from Bcat than from Wt mice (Table S2 and data not shown). Collectively, these results suggested that AOM-induced somatic mutations that further activate the Wnt/β-catenin pathway (i.e. gain-of-function mutations in *ctnnb1*; loss-of-function mutations in *Apc*), rather than mutations in the Ras-Erk signaling cascade, co-operate with ΔN-Bcat to trigger intestinal tumorigenesis.

### Level of tumorigenesis differs in mouse models for colorectal cancer

We previously showed that concomitant mutations in *Apc* and *Kras* increased intestinal tumorigenesis and mortality of compound pVillin-*Kras*^V12G^;*Apc*^1638N^ mice, where the *Apc*^1638N^ allele alone, upon loss of heterozygosity of the remaining wild-type *Apc* allele, confers development of three or four tumors per mouse (Janssen et al., 2006). Similar observations have also been reported in *Kras*^V12G^ compound mutant mice based on the stronger *Apc*^Min^ allele (Luo et al., 2009). We reconciled these observations, at least in part, by the ability of oncogenic *Kras*^V12G^ to enhance accumulation of nuclear β-catenin and hence to activate canonical Wnt/β-catenin signaling further. In order to gain a better understanding of the molecular mechanisms underlying the functional discrepancy between co-operations of *ΔN-Bcat* with the AOM-induced *Apc* and *Kras* mutations, respectively, we generated compound mutant pVillin-*Kras*^V12G^;*gpA33*^ΔN-Bcat^ mice (referred to as Ras/Bcat), *Apc*^1638N^;*gpA33*^ΔN-Bcat^ mice (referred to as 1638N/Bcat) and *Apc*^Min^;*gpA33*^ΔN-Bcat^ mice (referred to as Min/Bcat). Consistent with our results from the AOM challenge, we observed that the homozygous *ΔN-Bcat* allele conferred a small additive effect in the compound Ras/Bcat mice, yielding an average of four tumors per mouse compared with 1.7 and 2.3 tumors observed in the corresponding single mutants (Table 1). By contrast, *ΔN-Bcat* conferred synergistic effects on both *Apc* mutant alleles, averaging 7.8 tumors per 1638N/Bcat mouse and 62.5 tumors per Min/Bcat mouse compared with 3.9 tumors per Apc^1638N^ and 22.3 tumors per Apc^Min^ mouse. There were no gross morphological and histological differences between the lesions of the compound mutant mice, which were mainly well-differentiated adenomas (Fig. S3). Likewise, overall tumor incidence remained independent of the presence of the *ΔN-Bcat* allele (Table 1). Thus, *ΔN-Bcat* promotes intestinal polyposis in *Apc* mutant backgrounds in a synergistic manner, whereas it merely shows additive effects in mutant *Kras* animals.

**Table 1.**
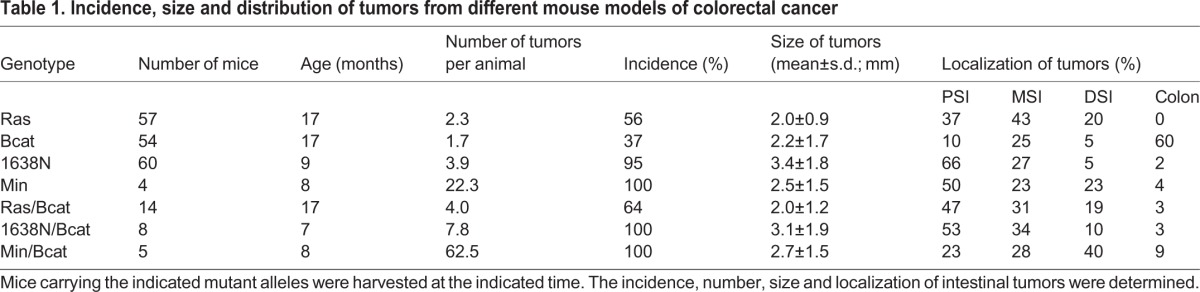
**Incidence, size and distribution of tumors from different mouse models of colorectal cancer**

### Establishment of a pro-angiogenic environment in the intestines of Bcat mice

**Fig. 5.**
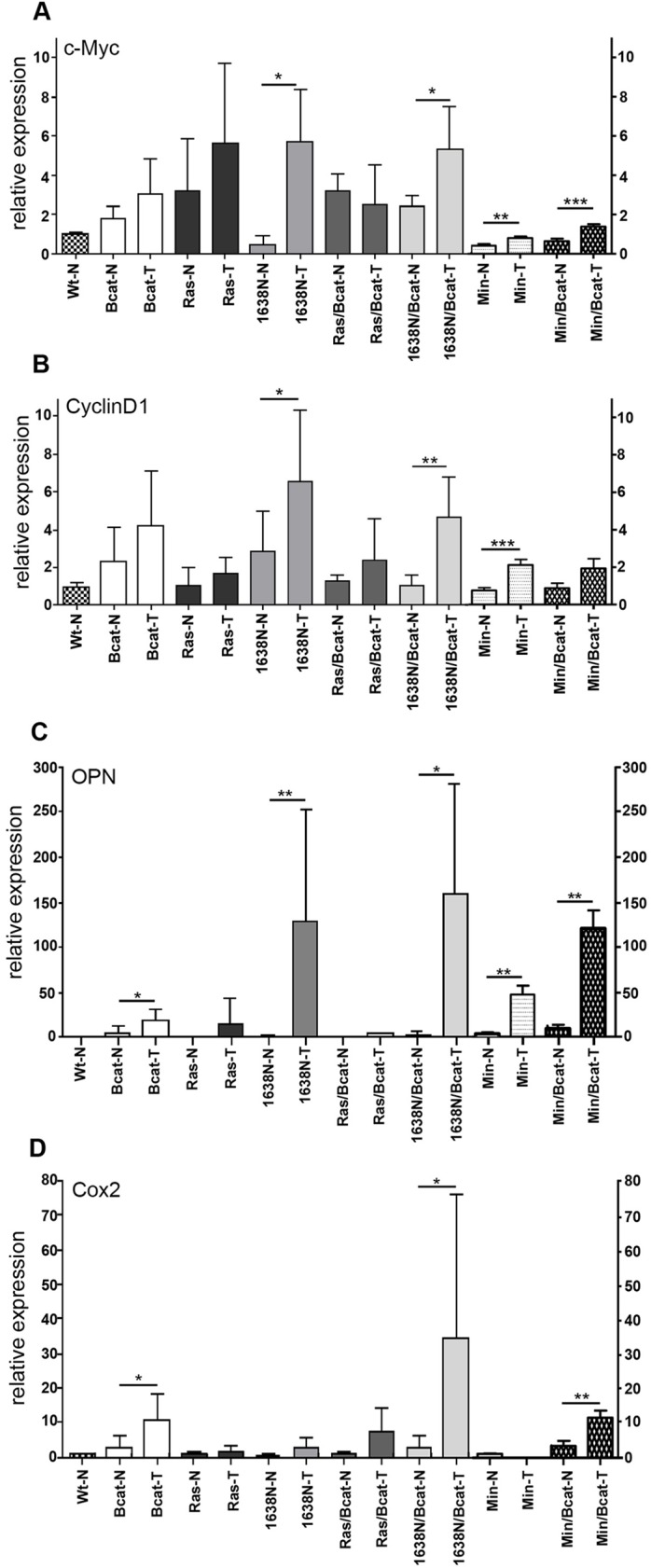
**Regulation of selected Wnt target genes in tumors of mice of the indicated genotype where no difference in proliferation is detected upon expression of the** ***ΔN-Bcat*** **transgene.** (A,B) RT-qPCR analysis of Myc and cyclinD1 mRNA expression in normal (N) and tumor (T) tissue from mice of the indicated genotypes (*P<0.05, **P<0.01, ***P<0.001; *n*≥5 tumors). (C,D) RT-qPCR analysis of osteopontin (OPN) and cyclooxygenase 2 (Cox2) mRNA expression in normal (N) and tumor (T) tissue from mice of the indicated genotypes (*P<0.05, **P<0.01; *n*≥5 tumors).

**Fig. 6.**
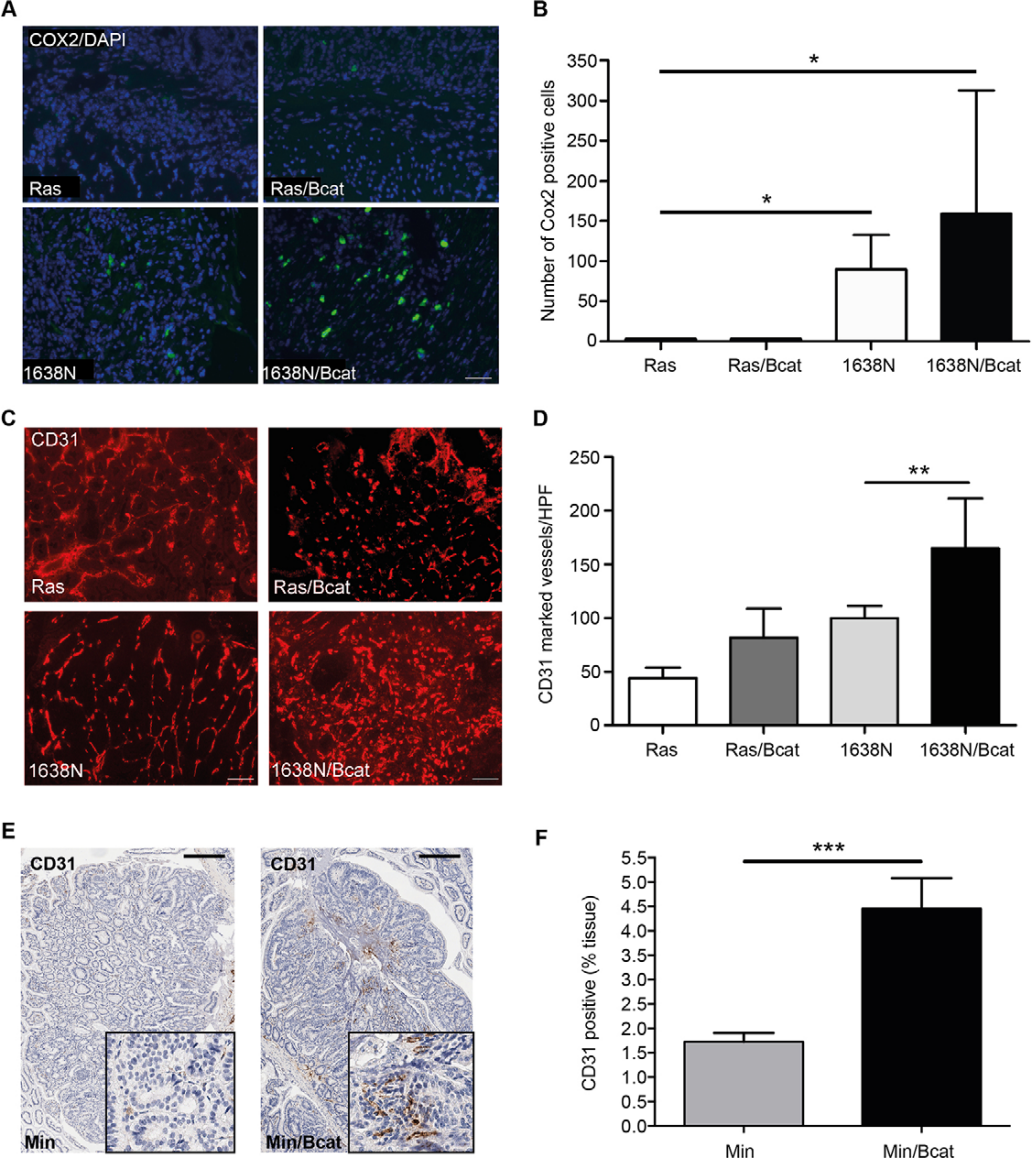
**Increased tumor angiogenesis in Bcat mice.** (A) Representative immunohistochemical Cox2 staining of tumor sections of the indicated genotypes (100× magnification, scale bar=100 µm). (B) Enumeration of Cox2^+^ cells per high-power field (HPF) of view in tumor sections from mice of the indicated genotypes. (*P<0.05; 10 HPFs counted per mouse and *n*>3 mice per genotype). (C) Immunofluorescence of cryosections stained with anti-CD31 antibody on tumors from mice of the indicated genotypes (100× magnification, scale bar=100 µm). (D) Quantification of CD31-positive vessels per HPF (**P<0.01; 10 HPFs counted per mouse and *n*>3 mice per genotype). (E) Representative immunohistochemical CD31 staining of cells in intestinal tissue sections of Min and Min/Bcat mice. (20× magnification, scale bar=100 µm; tumor inset: 200× magnification). (F) Quantification of CD31-stained area in tumours from Min and Min/Bcat mice (***P<0.001, *n*=3 mice per genotype).

In order to gain a better understanding of the molecular mechanisms that are likely to underpin the effects of the *ΔN-Bcat* mutation on intestinal tumor multiplicity, we monitored expression of the Wnt/β-catenin target genes *myc* and *Ccnd1*, which serve as a gatekeeper in Apc-dependent tumor formation (Sansom et al., 2007) and promote cell cycle progression, respectively. Surprisingly, expression of *myc* and cyclinD1 in tumors remained unaffected irrespective of whether they also harbored the *ΔN-Bcat* mutation and irrespective of whether they were associated with the mutations in *Kras* or *Apc* (Fig. 5A,B). Consistent with this, the average tumor size remained similar upon addition of the *ΔN-Bcat* mutation (Table 1). However, in the lesions of Min/Bcat compared with the other mouse models, the *ΔN-Bcat* allele conferred a strikingly increased expression of osteopontin and Cox2 (cyclooxygenase 2) (Fig. 5C,D), which can be considered as surrogate markers for activation of the Wnt/β-catenin pathway and inflammation (Araki et al., 2003; Mitra et al., 2012). We confirmed these findings by immunofluorescent staining of Cox2, which revealed significantly more Cox2^+^ cells in tumors of Ras/Bcat and 1638N/Bcat mice than in tumors of their single *Apc* and *Ras* mutant counterparts (Fig. 6A,B). Strikingly, the Cox2 staining was more prominent in the tumor stroma than in the epithelial tumor compartment. Given that Cox2 activity has been linked to increased tumor angiogenesis, we also stained tumor sections with the endothelial cell marker CD31 and observed a significant increase in CD31^+^ blood vessels in tumors of 1638N/Bcat and Min/Bcat mice, but not in tumors of Ras/Bcat mice (Fig. 6C-F). Collectively, these observations suggest that the *ΔN-Bcat* mutation co-operates with *Apc* mutations, which further activate the Wnt/β-catenin pathway to help in establishing a pro-angiogenic environment conducive to intestinal tumorigenesis.

## DISCUSSION

We have generated a new *gpA33*^ΔN-Bcat^ knock-in mouse model to study intestinal tumor susceptibility by inserting the 1-131 amino-terminal truncation mutant of β-catenin into the 3′ UTR of the endogenous *gpA33* antigen locus. Expression of this *ΔN-Bcat* transgene *per se* resulted in the formation of primarily colonic tumors in 37% of all Bcat mice of at least 8 months of age, akin to the long latency and low penetrance observed in human sporadic CRC. Our observation that *ΔN-Bcat* expression co-operates functionally with loss-of-function mutations in *Apc* rather than gain-of-function *Kras* mutations implies the existence of a minimal threshold level for canonical Wnt/β-catenin signaling to trigger tumor formation (Roose et al., 1999; Samuel et al., 2009; Albuquerque et al., 2010; Buchert et al., 2010; Leedham et al., 2013). Indeed, in Bcat mice we detected transcriptional activation of only a subset of Wnt/β-catenin target genes, including some specific for Paneth cells. This is consistent with models suggesting that different gene promoters require different levels of Wnt/β-catenin pathway activation for efficient transcription (Darken and Wilson, 2001; Albuquerque et al., 2010) and with mathematical calculations that the combination of gene-specific regulatory mechanisms with gradients of β-catenin and Apc functions are sufficient to confer distinct target gene expression patterns (Benary et al., 2013).

Functionally, Paneth cells contribute to intestinal homeostasis by providing niche factors to retain the stemness of Lgr5^+^ intestinal cells. Whereas the latter population also contributes the cells of origin for intestinal tumors (Barker et al., 2009), human colonic neoplasms are frequently characterized by excessive abundance of Paneth(-like) cells (Joo et al., 2009). Likewise, the serum concentrations of Tenascin-C and MMP7 are increased in individuals with CRC and have been proposed as biomarkers for primary and metastatic CRC, respectively (Pryczynicz et al., 2013; Takeda et al., 2007). Moreover, Tenascin-C is increasingly recognized to play an important role in shaping the tumor microenvironment (Spenlé et al., 2015). However, we noted that the increase in Paneth cells in Bcat mice was not accompanied by an increase in the stem cell markers Lgr5, Sox9 or Ascl2 in the normal intestine, nor activation of the Notch pathway (Fig. S4A-C, and data not shown), which also regulate cell fate decisions in the intestine. Likewise, in Bcat mice, we did not observe the increase in progenitor cell proliferation characteristically observed when the Wnt/β-catenin pathway is maximally stimulated following biallelic *Apc* inactivation (Sansom et al., 2006).

Several studies have reported the effects of intestinal expression of N-terminal mutants of β-catenin *in vivo*. The most extensive intestinal hyperproliferation and adenoma formation resulted from Cre-mediated excision of exon 3 (encoding amino acids 5-80) of the endogenous *ctnnb1* gene throughout the intestinal mucosa, thereby deleting all the regulatory serine and threonine residues that control the turnover of the β-catenin protein (Harada et al., 1999; Leedham et al., 2013). By contrast, mice that transgenically expressed β-catenin lacking amino acids 1-89 (Wong et al., 1998) remained tumor free at 10 months of age, but developed abnormal villus branching in the small intestine consistent with the rostrocaudal gradient of *Fabpl* gene promoter used to drive the transgene. Meanwhile, ubiquitous expression of a doxycycline-inducible version of the same β-catenin transgene resulted in rapid expansion of the intestinal crypt compartment, mislocalization of Paneth cells and upregulation of many Wnt target genes (Jarde et al., 2013). Transgenic expression of ΔN131β-catenin under the control of the calbindin promoter resulted in intestinal tumors strictly confined to the small intestine and premature death associated with transgene-induced polycystic kidney disease (Romagnolo et al., 1999). Here, we showed that expression of the same version of β-catenin, albeit as a homozygous knock-in transgene and under the control of the *gpA33* locus, resulted primarily in colonic tumors without detrimental effects in other organs. Notwithstanding the different nature of the various β-catenin truncation mutations in the above models, we interpret the different biological outcomes primarily as a consequence of the different spatial expression patterns, conferred by the various gene promoters, and ‘signaling strength’ as a function of the gene promoter and the nature of the β-catenin mutation. For instance, in their doxycycline-inducible model, Jarde et al. (2013) noted that transgene expression exceeded that of the endogenous *ctnnb1* gene by up to 11,000-fold, whereas in our Bcat mice the level of *ΔN-Bcat* expression remains more comparable to that of the simultaneously expressed endogenous wild-type protein. It remains to be determined whether the presence of the amino-terminal FLAG-tag and the associated introduction of bulky amino acids immediately following the 3′-end of the IRES account for moderate expression from the second cistron of the *gpA33*-IRES-*ΔN-Bcat* RNA (Bochkov and Palmenberg, 2006). Thus, in general, activation of the Wnt/β-catenin pathway in Bcat mice falls short of reaching the threshold required to induce adenoma formation in the colon, but predisposes these mice to tumorigenesis upon exposure to mutant *Apc* alleles or AOM-induced mutations in components of the Wnt signaling pathway. Bcat mice are therefore likely to provide a background that is genetically sensitized for the functional detection and confirmation of mutation variants in components of the canonical Wnt/β-catenin pathway.

Changes to intestinal permeability have recently been recognized as a contributing factor to intestinal mutagenesis. Here, we showed that relatively modest activation of the Wnt/β-catenin pathway increased intestinal permeability prior to formation of adenomas, and the former is likely to account for the increased expression of the pro-inflammatory proteins Cox2, osteopontin, IL-17 and IL-23.

We speculate that this might result from the impaired interaction of ΔN-Bcat with α-catenin, which is likely to weaken the interaction of the actin cytoskeleton with the plasma membrane in intestinal epithelial cells. Our observations suggest that oncogenic activation of the canonical Wnt/β-catenin pathway might help to set up a pro-tumorigenic microenvironment that precedes subsequent neoplastic transformation of the epithelium. This might be further exaggerated once tumors are established, given that the leakiness in *Apc* mutant colonic adenomas in mice triggered further accumulation of IL-17 and IL-23 in the tumor stroma (Grivennikov et al., 2012). Interestingly, cytoplasmic and nuclear β-catenin accumulation is detected in the majority of pre-neoplastic intestinal epithelium of individuals with inflammatory bowel disease (Claessen et al., 2010; van Dekken et al., 2007). Meanwhile, the importance of the IL-17/IL-23 axis is well documented for the pathogenesis of Crohn's disease and ulcerative colitis in humans (Clevers, 2006), and Cox2-derived lipids, including prostaglandin E_2_, are potent inflammatory mediators that promote tumor growth and metastasis (Wang et al., 2005; Xia et al., 2012). Conversely, for the non-transformed epithelium it is intriguing to speculate that the Wnt-signaling-dependent increase in Paneth cells and their transcripts is part of the response by the epithelium to increased bacterial antigen exposure. Upregulation of cryptidin 1 in the intestine of Bcat mice, for instance, is consistent with the upregulation of α-defensin 5 and 6 in the leaky and inflamed colons of individuals with ulcerative colitis (Fig. S5).

In conclusion, the Bcat mice provide a model that mimics some of the critical aspects of sporadic CRC induction in humans in terms of tumor multiplicity, tumor latency and tumor site specificity, and coincides with upregulation of the earliest markers for emerging CRC in humans, including Tenascin-C, MMP-7, osteopontin and COX2.

## MATERIALS AND METHODS

### Animal models

All experiments on mice were performed in accordance with institutional and national guidelines and regulations. Mice were maintained by crossing to C57Bl/6J animals. To control for genetic background effects, littermates were always used as controls. Mice were maintained under a 12 h:12 h light:dark cycle and fed with standard diet and water *ad libitum*. The *Apc*^Min^, *Apc*^1638N^ and pVillin-*Kras*^G12V^ models have been published previously (Janssen et al., 2006; Smits et al., 1998; Su et al., 1992).

### Generation of the *gpA33*^ΔN-Bcat^ mouse

The *gpA33*^ΔN-Bcat^ mouse (hereafter referred to as ‘Bcat’) was generated from a knock-in gene targeting vector comprising a lox(P)-flanked IRES-neo cassette and a cDNA in which the FLAG-epitope tag replaced the most amino terminal 131 amino acids of mouse β-catenin. The IRES sequence used is a modified version of the 5′ UTR from encephalomyocarditis virus (EMCV) mRNA (Mountford et al., 1994). The vector (Orner et al., 2002) also contained flanking sequences homologous to the last coding exon of *gpA33* and its 3′ UTR in order to capture all the *cis*-acting regulatory elements that collectively specify and confine expression of the resulting bicistronic RNA to the intestinal epithelium (Catimel et al., 1996; Heath et al., 1997; Johnstone et al., 2000). A lox(P)-flanked neo cassette provides a transcriptional roadblock for expression of the *ΔN-Bcat* transgene in *gpA33*^Neo^ mice. However, upon Cre-recombinase-mediated excision of the neo cassette following mating of female A33^Neo^ mice with male Cre-deleter mice, the modified *gpA33* locus of Bcat mice encodes a bicistronic RNA simultaneously encoding gpA33 and the FLAG-tagged ΔN(1-131)β-catenin (Orner et al., 2002). The 3′ end of the IRES and the 5′ ATG of the FLAG-tagged ΔN(1-131)β-catenin cDNA are separated by a short 13-bp linker region (5′-GCTTGCCACAACC-3′). The targeting vector was electroporated into W9.5 ES cells (129X1/SvJ), and correctly targeted ES cells were injected into blastocysts derived from C57/Bl6 donor female mice. Chimeric male offspring were mated with C57/Bl6 female mice and, once germ line transmission of the transgene was confirmed, the mice were backcrossed for at least 10 generations onto a C57/Bl6 background.

### RNA isolation

We isolated RNA from snap-frozen samples stored at −80°C. The RNA was isolated using the Qiagen RNeasy Kit (Qiagen, Hilden, Germany), and RNA integrity was confirmed on a denaturing formaldehyde-agarose gel. Preparation of cDNA was performed according to standard procedures, using RevertAid H-minus M-Mulv Reverse Transcriptase, random primer and oligo dT primers (Fermentas, St Leon-Rot, Germany).

### Quantitative real-time PCR

Quantitative real-time PCR was performed with the ABI PRISM 7300 detection system (Applied Biosystems, Foster City, CA, USA) using SYBRGreen dye. Relative RNA abundance was calculated using the ΔΔCT formula and normalized to the transcript levels of the housekeeping gene β-actin with the help of the Sequence Detection Software v.1.4 (Applied Biosystems). Assays were performed in duplicate. TaqMan primers for IL23 and IL17 were purchased from Life Technologies (Mulgrave, Victoria, Australia). Primer sequences used for SybrGreen RT-qPCR were as follows: β-actin/for, 5′-AGCCAGGTCCAGACGCAGG-3′; β-actin/rev, 5′-ACCCACACTGTGCCCATCTAC-3′; β-catenin/for, 5′-GCTGACCTGATGGAGTTGGA-3′; β-catenin/rev, 5′-GCTACTTGCTCTTGCGTGAA-3′; CD44/for, 5′-GTCTGCATCGCGGTCAATAG-3′; CD44/rev, 5′-GGTCTCTGATGGTTCCTTGTTC-3′; Cmyc/for, 5′-TAGTGCTGCATGAGGAGACA-3′; Cmyc/rev, 5′-GGTTTGCCTCTTCTCCACAG-3′; Cox2/for, 5′-ACACACTCTATCACTGGCACC-3′; Cox2/rev, 5′-TTCAGGGAGAAGCGTTTGC-3′; Cryptdin1/for, 5′-AAGAGACTAAAACTGAGGAGCAGC-3′; Cryptdin1/rev, 5′-CGACAGCAGAGCGTGTA-3′; CyclinD1/for, 5′-GCACAACGCACTTTCTTTCCA-3′; CyclinD1/rev, 5′-CGCAGGCTTGACTCCAGAAG-3′; ΔN-Bcat/for, 5′-GGATTACAAAGACGATGATGACAAGTTG-3′; ΔN-Bcat/rev, 5′-GTCAGCTCAGGAATTGCACGTG-3′; MMP7/for, 5′-GAGATGTGAGCGCACATCAGTG-3′; and MMP7/rev, 5′-GATGTAGGGGGAGAGTTTTCCAGT-3′.

### Human tissue samples

All samples were collected after prior informed written consent as part of a study (no. 1926/7) approved by the human ethics committee of the Klinikum rechts der Isar. Samples of histologically confirmed normal colonic mucosa from resected specimens (*n*=8) and from benign adenomas (*n*=9) were also analyzed. None of the individuals received neoadjuvant treatment or suffered from a known second neoplastic disease. Tumors were classified according to the UICC/TNM system (7th edition): UICC stage I (*n*=11 cases), stage II (*n*=10), stage III (*n*=8) and stage IV (*n*=13). The median of histologically reviewed lymph nodes per case was 21 (range, 7-72). Tissues from all 42 patients who underwent surgical resection between 1987 and 2006 at the Klinikum rechts der Isar were obtained immediately after surgical resection. Specimens were transferred into liquid nitrogen and stored at −80°C.

### Immunofluorescence on tissue sections

Cryosections of mouse tissues embedded in acetate buffer (AlleMan Pharma GmbH, Rimbach, Germany) were cut at 7 µm thickness, air dried and processed by routine hematoxylin and eosin staining. Some tissue sections were fixed in 10% normal buffered formalin overnight, processed and embedded in paraffin. For immunofluorescence, sections were fixed with either 3% paraformaldehyde at room temperature for 20 min or with methanol at −20°C for 10 min. The paraformaldehyde-fixed sections were treated with 50 mM NH_4_Cl in PBS for 20 min and solubilized with 0.1% Triton X-100 for 5 min. Antibodies used were as follows: anti-α-catenin (Abcam, Cambridge, UK), anti-β-catenin (BD, Franklin Lakes, NJ, USA and Sigma-Aldrich, St Louis, MO, USA, cat#C2206), anti-E-cadherin (Invitrogen, Camarillo, CA, USA, cat#13-1900, clone ECCD-2), anti-Cox2 (Santa Cruz, Heidelberg, Germany), anti-CD31 (PECAM1; Sigma-Aldrich, Munich, Germany), anti-Ki67, anti-Lysozym (Dako, Hamburg, Germany) and the dyes 4,6-diamidino-2-phenyl indol (DAPI; Sigma, Munich, Germany), TRITC-Phalloidin (Sigma) and TRITC-UEA1 (Sigma). Immunofluorescent staining of mouse tissue sections was detected with a Zeiss Axiovert 200M microscope with an AxiocamMR3 camera, with the following objectives: LD A-Plan 10×/NA:0.25, LD A-Plan 20×/NA:0.30 and Plan Neofluoar 40×/NA:0.75 lenses (all lenses: air, no immersion liquid). The tissues were imaged using standard filter sets and laser lines, acquiring single-labeled images. DAPI, FITC and Cy3 fluorescence were excited with a HXP120C lamp with filters at excitation wavelengths of 360, 490 and 550 nm, respectively, and the emission was measured at wavelengths 460, 520 and 562 nm, respectively. The images were captured using Zeiss Axiovision software (version 4.8.2). The Zeiss image files (.zvi) were imported into the Adobe Photoshop version 12.0.4 software for processing and display.

For immunofluorescent staining of paraffin-embedded tissue, sections were dewaxed in xylene and rehydrated. Antigen retrieval was in boiling 10 mM citrate buffer pH 6.0 for 15 min. Primary antibodies were diluted in 5% normal goat serum/0.5% Triton X-100 in PBS and incubated overnight at 4°C. Secondary antibodies used were goat anti-mouse IgG, goat anti-rat IgG and goat anti-rabbit IgG coupled to Alexa488, Alexa546 or Cy3 (Molecular Probes, Eugene, OR, USA). Tissues were mounted in ProLong Antifade plus DAPI (Life Technologies). Immunofluorescent staining of the distal colon was detected with a Leica SP8 Confocal microscope with Resonant Scanner (C4.50) on a 10× water immersion (NA 0.40) lens. The tissues were imaged using standard filter sets and laser lines, acquiring single-labeled images. DAPI and α-catenin fluorescence were excited with the 405 and 488 nm laser lines, respectively, and the emission was measured at wavelengths of 405 and 473 nm, respectively. The images were captured using Leica LAS-AF software. The Leica image files (lif) were imported into the ImageJ/Fiji software (Schindelin et al., 2012) for processing and display.

### Three-dimensional confocal fluorescence imaging

Immunofluorescent staining of crypts was detected with an Olympus FV1000 Spectral Confocal attachment to an Olympus IX-81 microscope on a 60× water immersion lens (NA 1.2). The crypts were imaged using standard filter sets and laser lines, acquiring single-labeled images. DAPI, β-catenin and E-cadherin fluorescence were excited with the 405, 488 and 546 nm laser lines, respectively, and the emission was measured at wavelengths of 405, 473 and 559 nm, respectively. The images were captured using Olympus FluoroView software (version 1.7c). Three-dimensional (3D) image stacks were acquired, which encompassed the entire depth of the crypt(s) in the field of view. The entire depth of the sample was acquired as 3D image stacks at approximately 20 µm thickness for each optical section. For quantitative spatial analysis of key proteins in isolated crypts, cubic voxels were acquired for each image stack. The output analog signal, representing the fluorescence intensities, was digitized to 16 bits resolution at 65,536 levels of gray and saved as an Olympus Image Binary (OIB) image. The OIB image files from the fluorescently stained individual whole-mount crypts were imported into the ImageJ/Fiji software (Schindelin et al., 2012) for processing and display. 3D image stacks were extracted and imported as ‘tiff’ files into MATLAB (MathWorks, Natick, MA, USA) for analysis. Quantification of β-catenin and E-cadherin was conducted as described by Tan et al. (2013).

### Preparation of protein lysates

Protein lysates were prepared from tissue samples stored at −80°C. Samples were homogenized in a Dounce Homogenizer (Wheaton, Melville, NJ, USA) with RIPA buffer [50 mM Tris-HCl, pH 7.5, 150 mM NaCl, 1 mM EDTA, 1% NP-40, 0.25% sodium-deoxycholate, 0.1% SDS and protease inhibitor cocktail (Roche, Mannheim, Germany)]. Soluble proteins were extracted after a 15,000 ***g*** centrifugation for 15 min at 4°C.

### Immunoprecipitation and immunoblotting

Protein G sepharose beads were incubated with 2 µg of anti-β-catenin, anti-FLAG or anti-E-cadherin antibody for 1 h at 4°C, and 40 µl of the Sepharose-antibody mixture was added to 400 µl cell lysate (see above, Preparation of protein lysates). After 2 h incubation, the samples were centrifuged at 15,000 ***g*** for 30 s at 4°C and washed three times with RIPA buffer.

For immunoblotting, protein lysates were separated by SDS-polyacrylamide gel electrophoresis under reducing conditions and transferred to a nitrocellulose membrane as previously described (Laemmli, 1970; Towbin et al., 1979). Immunoreactive bands were detected using anti-α-tubulin and anti-E-cadherin antibodies (Calbiochem, Darmstadt, Germany), anti-β-catenin (BD), anti-Apc (sc-896; Santa Cruz), anti-FLAG (Dianova) and anti-lamin antibodies (Cell Signaling Technology, Beverly, MA, USA). Secondary antibodies were horseradish peroxidase-conjugated goat anti-mouse IgG, goat anti-rabbit IgG or goat anti-rat IgG (Jackson Immunoresearch, West Grove, PA, USA), and bands were visualized with an enhanced chemiluminescence substrate detection kit (Pierce, Rockford, IL, USA).

### Isolation of cytosolic and nuclear fractions

Tissue samples were resuspended in ice-cold CLB buffer (10 mM Hepes, 10 mM NaCl, 5 mM NaHCO_3_, 1 mM CaCl_2_, 0.5 mM MgCl_2_, 5 mM EDTA and 1 mM Pefablock and protease inhibitor cocktail) on ice for 5 min, homogenized in a Dounce Homogenizer (Wheaton) and centrifuged at 1000 ***g*** (4°C) for 5 min. The cytosolic fraction (supernatant) was harvested after a centrifugation at 39,000 ***g*** for 15 min. The nuclear fraction (pellet) was resuspended in TSE buffer (10 mM Tris/HCl pH 7.5, 0.3 M sucrose, 1 mM EDTA, 0.1% NP-40, 1 mM Pefablock and protease inhibitor cocktail), homogenized and centrifuged at 1000 ***g*** (4°C) for 5 min. The pellet was resuspended in 100 µl RIPA buffer (see above, Preparation of protein lysates), and the samples were prepared for immunoblotting or immunoprecipitation.

### Tumor analysis and processing of tissue

Intestines were collected from mice at the indicated age and opened longitudinally to determine the size and location of macroscopically visible tumors, prior to their resection and embedding in acetate buffer (sodium acetate, sodium chloride, potassium chloride, calcium chloride and magnesium chloride hexahydrate, with respective molarities: 140 mM Na^+^, 4 mM K^+^, 2.5 mM Ca^2+^, 1 mM Mg^2+^, 106 mM Cl^−^, 45 mM acetate, pH 6.7-7.7; AlleMan Pharma GmbH, Rimbach, Germany) and processing for cryosections. Some freshly isolated tumors were snap-frozen in liquid nitrogen and stored at −80°C for subsequent DNA/RNA extraction or protein analysis. Aberrant crypt foci in the colonic mucosa of longitudinally opened, pinned out and fixed tissues (overnight in 75% ethyl alcohol, 20% formaldehyde and 5% acetic acid) were detected after staining for 1 min in 0.2% methylene blue (Sigma) in PBS and rinsed in fresh phosphate buffer at 4°C for 2 h. The tissue segments were placed with the luminal side up on microscope slides and observed with a low-magnification lens. All aberrant crypt foci were at least three times larger in diameter than normal crypts, and their lumina were mostly oval or elongated rather than circular (McLellan and Bird, 1988).

### Azoxymethane-induced mutagenesis

Eight-week-old mice were injected with 10 mg/kg AOM intraperitoneally once weekly for 6 consecutive weeks. Colons were collected either 5 weeks after the last AOM challenge to assess for aberrant crypt foci or at 12 weeks to assess for colonic adenomas. We used Sanger sequencing on genomic DNA prepared from adenomas to assess for AOM-induced amino acid substitution mutation K-ras (G12, G13, Q61) and β-catenin (S37, S38). Loss of heterozygosity was implicated by immunohistochemical absence of Apc protein staining using an antibody raised against the C-terminus of APC (sc-896; Santa Cruz).

### TRITC-dextran permeability assay

Intestinal permeability was assessed by gastric gavage of TRITC-dextran (40 mg/ml, 4 kDa in PBS; Sigma) as a non-metabolizable macromolecule (Brandl et al., 2009). Prior to the TRITC-dextran challenge (200 µl of a 40 mg/ml solution), we sensitized mice by providing 2% DSS in drinking water for 16 h. Measurements of TRITC-dextran in plasma were performed 4 h after the challenge using fluorometry.

### Statistics

Unless otherwise indicated, Student's *t*-test was performed and data were expressed as means±s.e.m.
